# Post-tuberculosis Aspergilloma in Undiagnosed Tetralogy of Fallot

**DOI:** 10.7759/cureus.2740

**Published:** 2018-06-05

**Authors:** Haseeb A Bhatti, Shueeta Kumari, Mohammad Hasan, Amber Siddiqui, Syed Maaz Tariq, Syed Ali Haider

**Affiliations:** 1 Department of Internal Medicine, Jinnah Postgraduate Medical Centre, Karachi, PAK; 2 Department of Medicine, Jinnah Postgraduate Medical Centre, Karachi, PAK; 3 Medical Student, Jinnah Sindh Medical University (SMC), Karachi, PAK; 4 Dow University of Health Sciences, Jinnah Sindh Medical University (SMC), Karachi, PAK; 5 Department of Medicine, Jinnah Sindh Medical University (SMC), Karachi, PAK

**Keywords:** tuberclosis, congenital heart defects, aspergilloma, primary aspergilloma, tetralogy of fallot, congenital heart defects

## Abstract

Tetralogy of Fallot (TOF) is the most common congenital heart disease (CHD) with an incidence of four in every 1000 live births in Pakistan. Classically, these children present with central cyanosis in early life; however, milder defects may remain asymptomatic for months or even years. We report a malnourished and anemic teenage male, who was admitted with shortness of breath, hemoptysis, fever, palpitations, and weight loss. On examination, vitals were stable, except for oxygen saturation, which was 84% on pulse-oximeter. Bilateral basal coarse crepitations were present on respiratory examination with a markedly reduced air entry in the right upper zone. A 2-3/6 systolic ejection murmur was appreciated on cardiac examination. The chest X-ray was consistent with a collapsed right upper lobe with fibrosis. Echocardiography was consistent with findings of TOF. Based on sputum for acid-fast bacilli (AFB smear) and GeneXpert (Cepheid Inc., Sunnyvale, California, US) Mycobacterium tuberculosis/resistance to rifampin (MTB/RIF), the patient was diagnosed with multi-drug resistant pulmonary tuberculosis (MDR-PTB). However, when the patient didn’t improve with anti-tuberculous therapy, a computed tomography (CT) scan chest was done, which raised a suspicion of aspergilloma. The culture and cytology of bronchoalveolar lavage (BAL) were done, which confirmed pulmonary aspergilloma. Undiagnosed congenital heart diseases are rare in adults. Pulmonary TB is rarely reported in right-to-left shunts; however, clinicians should maintain a suspicion of this correlation.

## Introduction

In Pakistan, the only reported incidence of congenital heart disease (CHD) is four in every 1000 live births [1]. One-quarter of all CHD reported is tetralogy of Fallot (TOF), making it the commonest CHD and the commonest cyanotic-CHD in Pakistan [2].

Classically, these patients have duct-dependent pulmonary circulation and become severely cyanotic immediately after birth, leading to early diagnosis and management. However, a subset of these patients, known as “pink TOF,” has mild duct defects and small shunts. These patients will not show any signs of circulatory instability for months or even years after birth [3].

## Case presentation

A sixteen-year-old male was admitted with complaints of shortness of breath and hemoptysis for three days. The patient also had complaints of palpitations, fever, and weight loss for two months.

On physical examination; he was a malnourished and anemic male, with a blood pressure of 130/80 mm of Hg, a pulse of 103 beats per minute, and oxygen saturation on pulse-oximeter of 84%. On respiratory examination, markedly reduced air entry in the right upper zone was noticed with bilateral basal coarse crepitations. A 2-3/6 systolic ejection murmur was appreciated on cardiac examination. Other systemic examinations were unremarkable.

**Figure 1:**
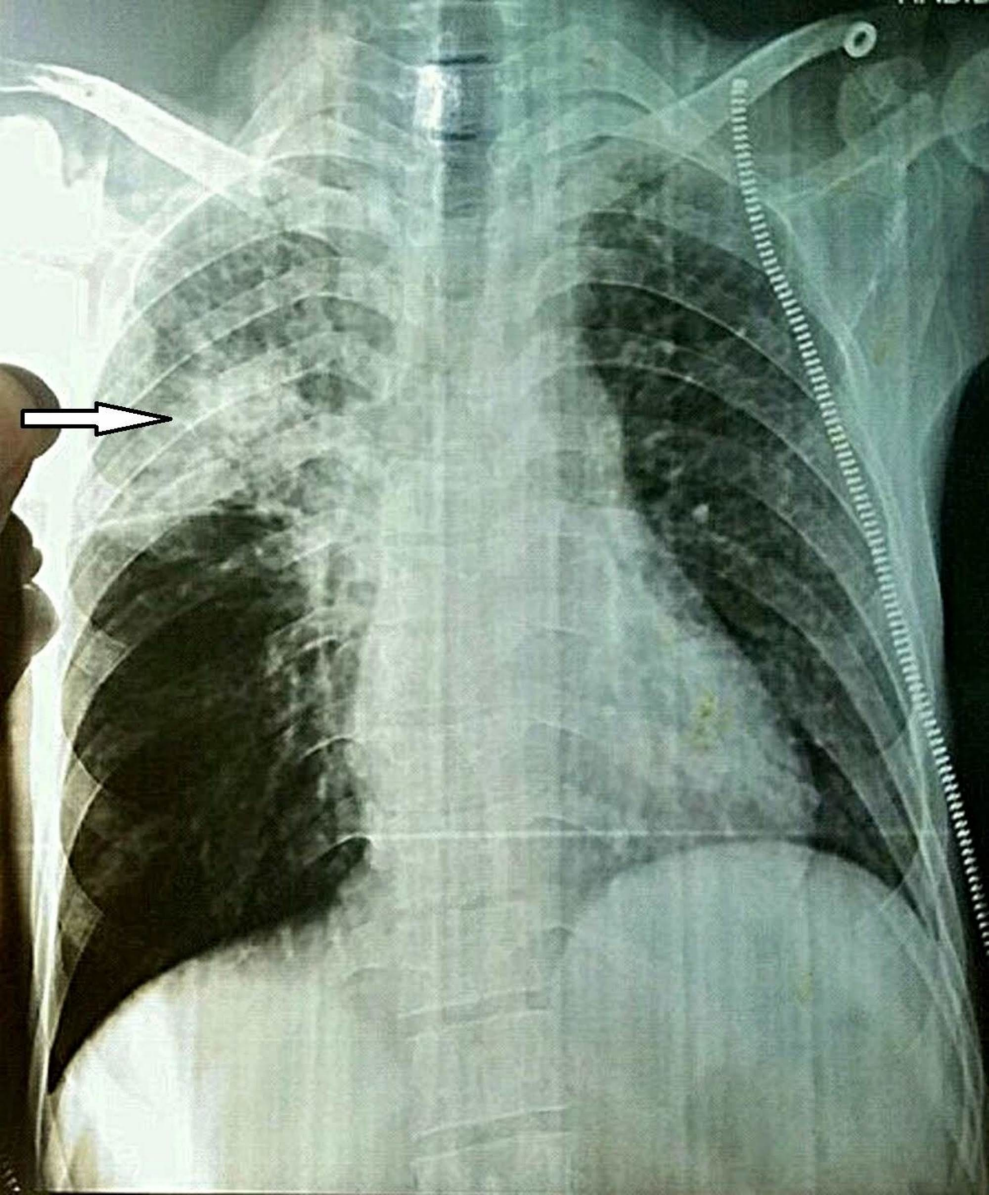
Chest X-ray shows boot-shaped heart with cavitation and fibrosis in the right upper lobe (arrowhead)

A chest X-ray (PA view) was done, which showed boot-shaped heart with cavitation and fibrosis in the right upper lobe, resulting in a collapsed right upper lobe (Figure 1).

A trans-thoracic echocardiographic study revealed an enlarged and hypertrophied right ventricle, a right-to-left shunt across the large ventricular septal defect with a mild overriding of the aorta, pulmonary stenosis, valvular as well as infundibular, and a right pulmonary artery of only 8 mm. The findings were consistent with tetralogy of Fallot (TOF). Sputum examination for acid-fast bacilli (AFB smear) was positive in two of three-morning sputum samples. GeneXpert MTB/RIF was also positive. Hence, the diagnosis of multi-drug-resistant pulmonary tuberculosis was formed.

The patient was started with anti-tuberculous therapy (ATT) with second-line agents, including injectable amikacin, along with oral levofloxacin, cycloserine, ethionamide, and pyrazinamide. All drugs were to be continued for 12 months; except for amikacin, which was to be stopped after eight months. All daily doses were adjusted according to the patient’s weight.

**Figure 2:**
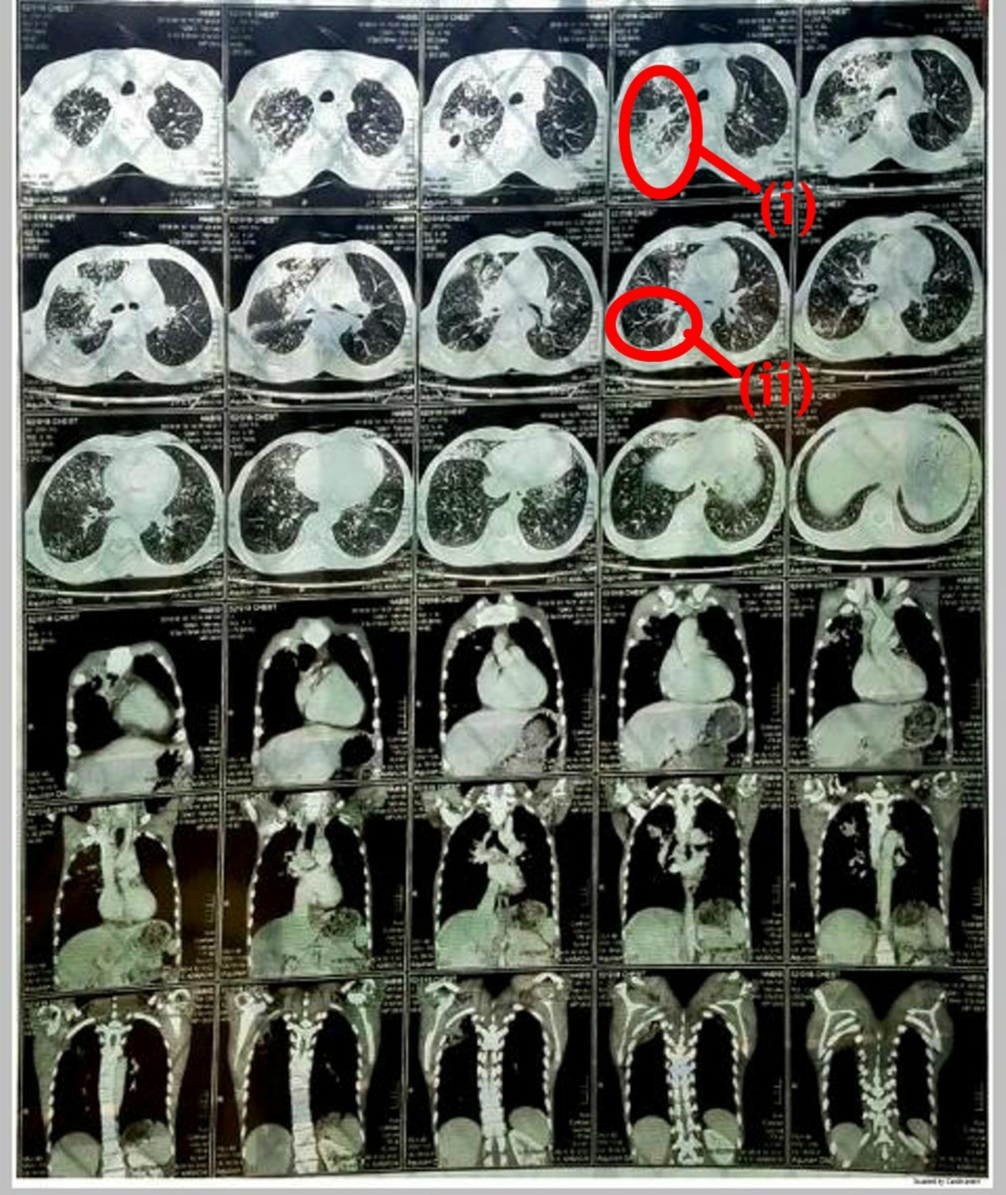
CT scan chest: (i) Multiple fluffy alveolar infiltrates; (ii) tree-in-bud appearance CT: computed tomography

However, the patient didn’t show any signs of improvements even after two weeks of ATT. A CT scan chest with contrast was then planned. It showed multiple fluffy alveolar infiltrates in both lung fields; some of which formed a tree-in-bud appearance representing an endobronchial spread. Patchy consolidation with cavitation in the right lung and middle right-sided pleural effusion with mediastinal lymphadenopathy were seen (Figure 2).

Based on the findings of CT chest, it was suspected that either tuberculosis had resulted in the endobronchial spread or there was another co-existing pathology. A bronchoscopy with culture and cytology for bronchoalveolar lavage (BAL) were done. Galactomannan (GM) was found in BAL fluid cytology and the culture was positive for Aspergillus species. It confirmed the presence of pulmonary aspergilloma.

Due to the non-availability of first-line drugs for aspergilloma–voriconazole and itraconazole–in this part of the world, the patient was started with amphotericin B, 50 mg intravenous (IV) once daily for 10 days. The patient was discharged on ATT and oral fluconazole 150 mg once daily for 21 days. Upon outpatient follow-up, the patient was seen to be considerably improving by the end of one month. BAL could not be repeated for GM, as the patient didn't consent. He is still on ATT and has been referred to a tertiary cardiovascular center for surgical repairment of his congenital heart condition.

We report this undiagnosed case of TOF with pulmonary complications secondary to immunosuppression for physicians practicing in third-world countries where tuberculosis is still endemic.

## Discussion

It is well known that pulmonary tuberculosis (PTB) is more common in patients with CHD. Van der Merwe et al. [4] reported PTB to be two and a half times more common in children with CHD. Patients with acyanotic CHD (with or without increased pulmonary blood flow) were reported to be more susceptible to developing pulmonary TB. No case of PTB was reported in patients with TOF, which is a cyanotic CHD with decreased pulmonary blood flow. Van der Merwe et al. explained that patients with cyanotic CHD have decreased pulmonary blood flow and cyanosis, which can inhibit the growth of M. tuberculosis whereas in acyanotic CHD, normal or increased pulmonary blood flow and normal pulmonary arterial saturation provides an encouraging environment for bacterial growth [4]. Ifere et al. have also reported two cases of PTB in presence of CHD other than TOF [5]. Olguntürk et al. reported PTB in a pediatric patient with tricuspid valve pathology, ASD, and pulmonary hypertension. The clinical suspicion was only confirmed on autopsy as all other investigations were negative [6].

To our knowledge, there are only two reported cases of pulmonary TB in the presence of TOF [7-8]. Radovic et al. [7] reported a middle-aged male patient with sputum positive TB who was investigated for CHD because of deteriorating cyanosis and respiratory insufficiency, even with anti-TB drugs. Echo was consistent with TOF. Gunay et al. [8] presented a young female, a diagnosed case of TOF, with respiratory complains, who was diagnosed with PTB and managed with first-line anti-TB drugs for two months. However, the literature doesn’t report any case of TOF with secondary PTB and secondary non-invasive pulmonary aspergilloma as in our reported case.

There are various diagnostic tools for pulmonary aspergilloma, including serum GM level, BAL GM, culture of Aspergillus species in sputum or BAL fluid, and serum Aspergillus precipitating antibodies. BAL GM assays were more sensitive than serum GM assays [9].

## Conclusions

Undiagnosed congenital heart defects rarely present in adults. Physicians dealing with PTB should keep a suspicion of underlying TOF in mind. Similarly, physicians dealing with known cases of TOF presenting with respiratory distress should also keep the differential diagnosis of pulmonary tuberculosis and aspergilloma in mind.

## References

[1] Hassan I, Haleem AA, Bhutta ZA (1997). Profile and risk factors for congenital heart disease. J Pak Med Assoc.

[2] Pate N, Jawed S, Nigar N, Junaid F, Wadood AA, Abdullah F (2016). Frequency and pattern of congenital heart defects in a tertiary care cardiac hospital of Karachi. Pak J Med Sci.

[3] Granbom E (2016). Respiratory tract infections in children with congenital heart disease [Article in English and Swedish]. DiVA.

[4] Van der Merwe PL, Kalis N, Schaaf HS, Nel EH, Gie RP (1995). Risk of pulmonary tuberculosis in children with congenital heart disease. Pediatr Cardiol.

[5] Ifere OA, Aikhionbare HA, Yusuf U (1989). Congenital heart disease masking pulmonary tuberculosis in children. East Afr Med J.

[6] Olguntürk R, Tunaoğlu FS, Gökgöz L, Memiş L, Kula S (2010). Congenital heart disease and pulmonary tuberculosis. Cent Eur J Med.

[7] Radović M, Ristić L, Stanković I, Petrović D (2010). Rare congenital heart disease as a cause of tuberculosis activation [Article in English and Bosnian]. Med Pregl.

[8] Günay E, Günay S, Karakuş G, Şahin T, Görgün D, Tursun I, Dural C (2012). Pulmonary tuberculosis in an adult patient with tetralogy of Fallot. Hippokratia.

[9] Park SY, Lee SO, Choi SH (2011). Serum and bronchoalveolar lavage fluid galactomannan assays in patients with pulmonary aspergilloma. Clin Infect Dis.

